# Supplementary material for: Are carbon emissions trading and green financial instruments synergistic? -Comprehensive quantitative research based on content analysis
Source: PLoS One. 2024 Mar 7;19(3):e0298601. doi: 10.1371/journal.pone.0298601 (PMC10919601; doi:10.1371/journal.pone.0298601)
Supplement: S1 Data — (ZIP) [file pone.0298601.s001.zip › Data/地级市Z_F工作报告.zip!/┴╔─■/▒╛╧¬╩╨/└┤╫╘╣1⁄2╓┌║┼í╛┬φ┐╦╩2╛▌═°í┐_2011.docx]

各位代表：

　 　现在，我代表市人民政府向大会报告工作，请予审议，并请市政协委员和各位列席同志提出意见和建议。

　 　一、2010年工作回顾

　 　2010年，在省委、省政府和中共本溪市委的正确领导下，在市人大、市政协的支持监督下，市政府团结带领全市人民，较好地完成了市十四届人大三次会议确定的目标任务。预计：全市生产总值实现850亿元，增长16.3%。地方财政一般预算收入完成75亿元，增长27.4%。规模以上工业增加值完成420亿元，增长18%。全社会固定资产投资完成500亿元，增长40%。社会消费品零售总额完成192亿元，增长18.5%。实际利用外资3亿美元，增长49%。出口创汇15亿美元，增长132%。引进内资440亿元，增长99%。全市金融机构各项贷款余额630亿元，增长27.8%。城镇居民人均可支配收入16470元，增长12%。农民人均纯收入7830元，增长16%。超额完成了“两个不低于”的奋斗目标。胜利实现了“十一五”规划确定的目标任务。过去的五年，是本溪经济实力提升最快的五年，是城乡面貌变化最大的五年，是人民群众得到实惠最多的五年，是本溪奠定坚实发展基础重要的五年。

　 　（一）重点产业集群培育取得新进展。国家医药产业基地已有193个项目签约入驻，签约合同投资额623亿元，达产后可实现销售收入980亿元。完成注册企业109家，开工项目102个，投产企业30家，研发机构运营22家,主体建成企业31家。基地成为国家级生物医药科技产业基地、国家创新药物孵化基地和省级生物医药高新技术产业开发区。本钢集团主导产品产量稳定增长，粗钢产量突破千万吨大关，工业增加值和销售收入分别增长31.6%和23.6%。钢铁深加工产业园获批建设省级园区，桥北和东风湖两个产业园核心区路网等基础设施开始启动。园区开工项目31个，储备项目30个。大台沟铁矿前期工作全面启动。本溪水洞温泉旅游度假区获批建设“辽宁山水旅游休闲基地”。总投资80亿元的温泉寺健康养生小镇、发家峪山地休闲公园等4个项目签约入驻。完成了15公里温泉寺旅游景观路工程。

　 　（二）沈本一体化建设再上新台阶。完成了沈本城际连接带建设规划和沈溪新城、太子河新城总体规划及部分控详规划。沈溪新城在全力推进医药产业基地加快建设的同时，规划建设了“一心、两带、三园、三城”等城市功能区。启动实施了中央大街、环城公路等新城基础设施建设，启动实施了核心区保障性安居工程，启动实施了会展中心、总部基地、低密度酒店等一批公共服务项目。新城博物馆、服务中心正在加快建设。新城规划展示馆、科学家会堂、仙榆湾酒店、研发中心等投入使用。围绕建设现代健康城市，签约引进了香港俊安、深圳朗钜、上海三盛、浙江旺力、东方园林等公用设施及房地产开发项目。太子河新城规划启动了新城中央大街、三家子大桥等路网工程，启动建设了新城核心区保障性安居工程，签约引进了上海绿地、深圳佳兆业、浙江中旭等一批城市综合体项目。

　 　（三）固定资产投资实现新攀升。能源交通方面，本溪热电、桓仁抽水蓄能项目加快推进。沈丹客专全线开工。田桓铁路项目可研获得批复。钢铁大道工程启动实施。工业方面，滚动推进400个工业技改项目，完成投资260亿元。新增规模以上工业企业87户。农业方面，雨润食品、汇源果汁、永祥羊绒等项目相继投产，张裕葡萄酒、长白仙子等一批项目进展顺利，新增规模以上农事龙头企业60户。全市房地产业完成投资65亿元，在建施工面积670万平方米。

　 　（四）县域经济发展开创新局面。全市粮食产量21.66万吨，做到遭灾不减收。中药材、山野菜、干坚果等林地产业新增18万亩。新建标准化养殖小区110个。建成现代农业示范基地37个。新增农民专业合作社100个。新建农村饮水安全工程53处。新建小型农田水利灌溉工程126项。粮食、农机、良种补贴6776万元，受益农户14.6万户。狠抓了工业产业集群建设，两县绿色食品、葡萄酒和新型材料等产业集群初具规模。两县结合建设30万人口的中等城市，房地产开发面积100多万平方米。两县生产总值平均增长24%以上，地方财政一般预算收入平均增长50%左右。

　　 （五）城区经济发展呈现新亮点。城区经济狠抓了工业产业园区和服务业集聚区，规划建设了7个工业产业园区、10个现代服务业集聚区，火连寨新型建材产业园、高台子汽车零部件产业园、南芬铸件产业园产业规模不断扩大。永丰商业、卧龙汽车、彩北物流等服务业集聚区发展态势良好。主城区十里金街项目开始启动。站前商业中心签约引进了站前不夜城项目，地工商务中心签约引进了红星美凯龙和欧洲城项目，北地金融中心签约引进了兴业、广发、浦发和盛京银行，大峪文体中心签约引进了朗钜国际文化城项目。

　　 （六）城市建设管理水平又有新提高。完成46条街巷路和“摸黑路”整治。初步完成重点区域供热管网改造。城区新栽树木25万株。新建绿地4.8万平方米。清理乱堆乱放2.2万处，清运建筑垃圾10.3万吨，拆除乱搭乱建4036处，查处违章停车2.1万辆次。完成1万座农村改厕项目。溪河路、彩屯路、育明路等续建工程加快推进。桓仁县荣获国家卫生县城。

　　 （七）生态环境建设再创新佳绩。完成绿化造林15.1万亩。启动沈丹高速公路、本桓公路等生态环境建设工程。公路“环境杯”竞赛活动取得阶段性成果。我市获得“国家森林城市”和“全国绿化模范城市”荣誉称号。搬迁改造本溪水泥厂，北钢三焦脱硫等替代项目顺利实施，污水处理厂建设全面完成，城市环境综合整治考评全省排名由第10位晋升到第4位。实施重点污染源治理和节能减排项目30项，实现减排二氧化硫0.24万吨、化学需氧量0.4万吨，基本实现“十一五”总量控制目标。单位生产总值综合能耗下降4.0%。环境空气质量二级（良）以上天数348天，一级（优）天数70天。

　　 （八）改革开放取得新突破。本钢与北钢实现合并重组。四环药业兼并本溪雷龙探索了引进企业重组改革的成功范例。集体林产权制度改革受到国家林业局肯定。进一步完善了市、县区财政体制。完成城投公司平台改造。新城基础设施投资新增15亿元。在重点产业园区实施了人事制度改革。扩大县区和产业园区经济管理权限开始实施。组织赴香港、温州、宁波、日韩等招商活动30余次。举办了首届辽宁本溪钢铁深加工招商推介会和医药产业侨商投资洽谈会。全年共签约引进各类项目579个，到位资金千万元以上项目408个、亿元以上项目83个。

　　 （九）民生工程取得新成效。实现城镇实名制就业8.2万人。劳务输出2万人。城镇登记失业率控制在3.99%。企业退休人员月人均增加养老金117元。全额救助城乡低保对象参加城镇居民医保和新农合。新型农村社会养老保险试点全面启动。城镇职工医保最高支付限额提高到6.2万元。新农合人均补助标准提高到120元，最高支付限额提高到4.5万元。失业保险人均月增加120元。城区职工月最低工资提高到750元。城乡低保人均救助额分别提高到236元和127.3元。新建灾民房屋183户，维修房屋1135户。为6000余户家庭核发廉租住房租赁补贴。新建农村特困户住房100套。发放社会救助金3103万元，惠及城乡困难群众15.4万户次。城乡贫困群众取暖救助3040万元。

　　 （十）社会事业建设取得新成果。本钢信息自动化公司获批国家级高新技术企业。全年开发新产品230项，实现销售收入360亿元。全市高新技术产品增加值完成92亿元，增长25.5%。教育事业全面发展。学前幼儿入园率89%，位居全省前列。义务教育成为全国均衡发展试点市。辽宁冶金职业技术学院正式挂牌。本溪电大被评为全国示范性基层电大。接纳安置672名青海玉树学生来溪学习。本溪湖中国近代煤铁工业遗址园列入国家第二批红色之旅经典景区名录。《家乡好》、《探亲》和《女人是座山》等文艺作品分获全国、东北和省级大奖。“本溪松花砚雕刻技艺”等12个项目申报省级非物质文化遗产。本溪图书馆被评为国家一级馆。市文化局获得全国文化系统先进集体。医药卫生体制改革深入实施。卫生资源整合经验在全省推广。新改扩建社区卫生服务中心和村卫生室21所。我市在第十一届省运会上获得青少年组金牌、奖牌、团体总分三项第四名的历史最好成绩。我市获得省“文明城市”称号。本溪市被国家确定为人口计生综合改革示范市。人口普查工作进展顺利。人防工作获得全国先进城市称号。史志办荣获全国方志系统先进集体。市档案馆晋升为国家二级档案馆。全市信访总量同比下降45%。安全生产排查治理各种隐患2393项，各类事故死亡人数同比下降14.8%，较好遏制了重特大生产安全事故的发生。社会治安综合治理被评为全省先进市。公安“三项建设”在全国推广。全市刑事案件和治安案件同比分别下降59.5%和51.8%，147个社区（村）实现了零发案，人民群众的安全感、公正感、满意度持续增强。外事、侨务、对台、民族、宗教、老龄、残联、双拥、地震、气象等工作也取得新的成绩。

　 　过去的一年，我们着力依法行政、为民执政、廉洁从政，切实加强了政府自身建设。坚持依法决策，规范行政行为，推进政务公开，加强了法治政府建设。提请市人大常委会制定地方性法规3件，制定市政府规章10件，清理政府规章98件、规范性文件278件。市政府和桓仁县政府被评为全省依法行政工作先进单位。自觉接受人大依法监督和政协民主监督。办理人大代表建议和政协提案1163件，办复率100%，解决率69%，满意率99%。市长公开电话和“市长信箱”受理市民投诉和信件分别为12887次（件）、3374件，办结率和回复率分别为99%和99.3%。不断强化行政监察和审计监督，查处各类问题资金11亿元。开展以信访稳定、服务环境和县区发展为内容的绩效考评，促进了政府职能转变。

　 　过去的一年，我们深知所取得的成绩，无不蕴藏着省委、省政府的正确领导和关心支持，无不体现着中共本溪市委贯彻科学发展观的正确决策，无不饱含着各级人大代表、政协委员的支持和帮助，无不凝聚着全市人民的心血和汗水。在这里，我代表市政府，向辛勤工作在各行各业的全市人民，向给予政府工作大力支持的各级人大代表和政协委员、各民主党派、工商联、无党派人士、各人民团体和社会各界人士，向驻溪中省直单位和驻溪解放军、武警部队官兵，向所有关心支持本溪发展的海内外人士表示衷心的感谢!

　 　过去的一年，我们在工作中还清醒认识到，保持本溪经济社会发展，保持本溪社会和谐稳定，还面临诸多困难、矛盾和问题：我市经济社会发展中的结构性和体制性问题仍然并存，经济社会转型和产业结构调整面临诸多制约，生产要素供给和资源环境约束更加明显，经济总量偏小和开放发展空间矛盾突出，城乡居民生活水平和一些发达地区相比差距较大，各级政府执行能力还不能适应激烈竞争发展的客观要求，加快改善民生和保持社会和谐稳定的任务仍然艰巨。对此，我们要在今后工作中着力加以解决。

　 　二、2011年主要工作任务

　 　“十二五”是本世纪的第三个五年规划。这五年，对于我们国家来说，仍然处在大有可为的重要战略机遇期；对于辽宁来说，将是经济社会迅猛发展的快速增长期；对于本溪来说，必将是“三都五城”建设的关键期；对于县区来说，一定是经济实力全面提升的高潮期；对于人民群众来说，应当是得到实惠更多、实现幸福感更大的新时期。

　 　市委十届十次全会明确了“十二五”全市经济社会发展要扩大经济总量、调整产业结构、建设宜居城市、提高人民生活水平的总体目标，提出了累计完成固定资产投资5000亿元，地区生产总值实现2000亿元，地方财政一般预算收入实现200亿元，城乡居民收入进入全省前列，整体发展水平逐步向全国地级城市百强迈进的奋斗目标。这是全市人心所向和利益所在，我们必须肩负起这个神圣的历史使命。

　 　新的一年，我们要深入贯彻落实全省、全市经济工作会议和市委十届十次全会精神，紧紧围绕“昂首十二五、建设新本溪”的总体要求，以科学发展、创新发展、和谐发展为主题，以转变经济发展方式和产业结构调整为主线，以沈本一体化为载体，坚持扩大增量调结构，盘活存量上规模，着力推进产业集群化、城市现代化和民生工程化，在确保“两个不低于”的基础上，使全市经济总量、发展动力、竞争实力和人民生活再上一个新台阶。

　 　新的一年，全市经济社会发展的预期和控制目标是：地区生产总值实现950亿元，增长13%。地方财政一般预算收入实现85亿元，增长15%以上。全社会固定资产投资实现750亿元，增长50%。社会消费品零售总额增长18%。出口创汇增长15%。实际利用外资和引进内资均增长30%。金融机构各项贷款余额新增200亿元。城镇居民人均可支配收入增长14.5%。农民人均纯收入增长15.5%。单位生产总值综合能耗下降4.0%。实现实名制就业6.5万人。城镇登记失业率控制在4.2%以内。居民消费价格总水平涨幅控制在全省平均水平以内。

　 　新的一年，市政府要重点做好以下工作：

　 　（一）加快推进产业结构优化调整。在全力支持本钢集团做强做大的同时，加快培育发展生物医药、钢铁深加工和旅游度假三个产业集群。国家医药产业基地要抓好企业投产、平台运营、项目引进，实现新增开工项目30个、建成投产项目30个、招商签约项目100个，医药产业引资额200亿元以上，加快提升中国药都新形象。启动辽宁新药创制中心。大力引进科技领军人才。积极申报国家级高新区、省级医疗器械产业园和国家级生物医药高新技术产业开发区。筹备召开辽宁（本溪）第三届生物医药高新技术交易会。辽宁（本溪）钢铁深加工产业园要完成入驻企业50户，签约储备项目100个，争取20户企业竣工投产。加快推进本钢三冷轧规划建设和北营厂区整体改造。全力支持大台沟铁矿和思山岭铁矿开发建设。加大对现有中小企业扶持力度。本溪水洞温泉旅游度假区要完成新签约项目10个以上。加快建设五星级酒店、温泉会馆、温泉小镇、山地运动公园、太子河水上乐园及旅游地产项目。基本完成区域内旅游景观道路、景观水系和保障性安居工程。

　 　同时，还要注重抓好葡萄酒、绿色食品、新型材料、地域文化等特色产业加快发展。

　 　（二）全力推动沈本城际连接带建设。沈溪新城要着眼建设东北亚现代健康城市、国家生物医药产业基地和沈阳经济区改革创新先行区，全力推进医药产业集群发展，完善提升药都大学城，开工建设药都商贸城，规划启动药都健康城。实施中央大街、高铁大街、高速大街和环城路网等基础设施建设。沈阳药科大学、中国医科大学、辽宁中医药大学本溪校区基本建成。推进会展中心、金融中心、人才中心和文体中心等公共平台建设。太子河新城要着眼建设本溪高端宜居新城、沈阳经济区都市休闲中心和东北亚旅游度假城市，重点推进深圳佳兆业水岸新城、上海绿地生态商务城、浙江中旭威尼斯水城等项目入驻建设。加快推进“四纵四横”新城路网体系和保障性回迁社区建设。

　 　（三）加快发展县域经济和现代农业。县域主要经济指标要按照“五年翻两番”的目标进行安排。推进城乡统筹配套改革试点工作。以增加农民收入为核心，推进农业产业结构调整。加大对“三农”的投入力度，抓好农业产业化。引进培育年销售收入超亿元的农事龙头企业30个。建设好现代农业示范带和冰葡萄、中药材、绿色蔬菜等现代农业示范区。加强蔬菜基地建设，抓好“菜篮子工程”。要抓好工业产业集群建设。两县要重点培育冰葡萄酒、绿色食品和新型材料产业集群。要抓好旅游业集聚区。本溪县重点建设汤河农家乐山庄群，桓仁县重点建设桓龙湖酒庄群。要抓好城镇规模化。两县县城要按照30万人口的中等城市规模规划建设。大力开展农村土地整治工作，启动建设田师付、华来等新市镇，规划建设一批中心村。完善以城带乡、统筹发展的强农惠农机制，抓好一批农村道路、农田水利、能源环保等项目建设，加快推进新农村建设。坚持开发式扶贫，全力提高低收入人群收入水平。

　　 （四）全力实施工业“五项工程”。强化对本钢集团生产经营、技术改造的政策扶持。抓好规模以上工业企业的运行，支持工业企业异地搬迁、技术改造和扩大生产经营。全市规模以上工业增加值要增长16.5%。全年新增销售收入超10亿元企业2户、超5亿元企业14户。继续滚动推进400个重点技改项目。实现企业并购4个以上。实行产业项目能耗准入制度。实施重大节能工程20项。积极争取国家、省专项资金，市本级筹集2000万元，用于企业兼并重组、并购上市、争创名牌和驰名商标等。加快用信息化推动工业化发展。大力发展建筑业，全市建筑业产值实现150亿元以上。

　 　（五）大力发展城区经济和现代服务业。城区经济要抓好工业产业集群和服务业集聚区建设。平山区重点打造桥北钢铁深加工产业园和站前中心商业区。明山区重点建设汽车零部件产业园和卧龙北方汽车服务园。溪湖区重点建设东风湖钢铁物流和火连寨新型建材产业园。南芬区重点建设铸件产业园和思山岭文化产业园。通过几年的努力，每个区都应有一个或几个超100亿元的工业产业集群和服务业集聚区。要抓好社区建设，深入开展文明、生态、康乐、和谐“四型”社区创建活动。要加快完成剩余采煤沉陷区、棚户区改造及城中村开发。以房地产、旅游度假、投资融资、商贸流通等产业为重点，不断增强消费对经济的拉动作用。全年住宅地产、工业地产、商业地产、旅游地产等新开工600万平方米以上，竣工300万平方米以上，实现销售收入200亿元以上。支持本钢建设集团、实华集团、华厦集团、建工集团等企业加快发展。注重引进培育国内外知名房地产开发企业。举办好第八届中国·本溪国际枫叶节。接待国内外游客及旅游总收入分别增长30%以上。继续实施“家电下乡”、“万村千乡市场工程”。加快银行、证券、保险、基金等高端金融业发展，加强信用环境建设。加快发展物流配送、电子商务、信息咨询、中介服务等现代经营业态。规范五金机电行业市场。规划建设若干个停车场及“二手车”交易市场。改善公共交通服务质量。加大食品药品等市场服务监管力度。强化价格监测，调控和稳定物价。

　　 （六）全力推动基础设施和宜居城市建设。推进本溪热电联产、桓仁抽水蓄能和“引观入本”工程开工建设。推进东边道铁路桓仁段、沈丹高铁本溪段、田桓铁路、沈本城际轨道交通等铁路项目的启动建设和竣工通车。推动东部高速桓仁段、桓抚高速、本桓高速、本抚高速等高速路的竣工通车和启动建设。推动沈本产业大道、沈本旅游大道、太子河景观路连接线开工建设。规划启动本溪主城环城公路、沈溪新城环城公路、桓龙湖环湖公路建设工程。推进主城区和新城区路网体系、水利工程、通信工程、电力工程等市政设施建设。实施主城区区域供暖改造。高标准规划建设观音阁新城、桓仁新城、桥北新城和南芬副城。加强节能减排和生态环境建设。实施本钢烧结机脱硫和本溪水泥厂环保搬迁等11项重点污染源治理工程。实施溪湖河综合整治等5项水污染防治工程。实施两县12个乡镇、70个村农村环境综合整治工程。精心维护好浑江、太子河等水系生态环境。继续开发利用好平顶山城市公园。积极开展森林“七进”活动，巩固国家森林城市创建成果。继续做好创建全国环保模范城市和国家卫生城市各项工作。启动创建国家园林城市。

　　 （七）着力推进改革开放。要加快推动各类企业的改革创新。支持本钢加快重组。注重各类企业用存量吸纳增量。注重通过招商推动各类企业兼并重组。注重鼓励民营经济加快发展。要特别注重全民创业。要加快推动政府改革创新。推进行政审批制度改革，提高办事效率。以拓展发展空间和理顺管理体制为目的，深化区划调整。推动市级部分经济管理权限下放到县区和产业园区。完善财税制度改革，加强财税管理和政府债务管理。推进文艺团体、图书出版发行、网络资源等文化体制改革。深化集体林产权制度配套改革。要加快推动要素市场改革创新。抓好银行贷款规模，包装好一批重大融资贷款项目。抓好企业上市工作，制定一系列鼓励政策，培育一批上市企业。抓好多渠道使用股权基金，采用BT、BOT、FOT等方式，推动城市基础设施加快建设。继续抓好城投平台和债券融资。抓好存量建设用地的开发利用，整合废弃工矿企业建设用地。抓好城中村开发改造，用集体建设用地解决建设用地不足问题。抓好城乡建设用地增减挂钩。抓好河滩地、废弃地等未利用地的开发利用。抓好新增建设用地指标的高效使用。培育引进一批善管理、懂经营、肯吃苦、作风好的骨干人才队伍。项目是集成资金、土地、人才等生产要素的基本载体，今年要启动实施“项目年”，围绕固定资产投资项目的包装设计、招商引资、审批服务、开工建设、竣工运营、技术改造等开展好一系列活动。牢固树立环境就是生产力的思想，坚决查处推诿扯皮、吃拿卡要等不作为、乱作为行为。推进诚信本溪建设，大力营造亲商、安商、富商的良好环境。

　　 围绕培育生物医药、钢铁深加工、旅游度假三个产业集群，围绕县区工业园区和新城新市镇建设，采取“走出去、请进来”等多种形式，针对日、韩、港、台等国家和地区，针对“珠三角”、“长三角”、“京津唐”等国内发达地区开展系列招商引资活动。要抓好对外开放的发展空间。各类产业园区、新城新市镇是最重要的发展空间，要抓好产业园区、新城新市镇的总体规划、征地搬迁、基础设施建设，确保招商引资项目落地开工。积极调整出口结构，确保出口创汇稳步增长。

　　 （八）统筹发展各项社会事业和着力改善民生。完善企业为主体的技术创新体系，全市高新技术产品增加值增长25%以上。积极发展教育事业。不断加强学前教育。继续促进义务教育均衡发展，改善薄弱学校办学环境，理顺初中管理体制。提升高中阶段教育质量，完成市高中分校建设。加快发展职业教育。依托药都大学城，推进高等教育蓬勃发展。大力发展文化事业和文化产业。规划建设辽砚文化产业园、本溪湖中国近代煤铁工业遗址园、庙后山远古人类遗址园等项目。启动市艺术宫开发改造。深化医药卫生体制改革，建立健全覆盖城乡居民的基本医疗卫生制度。深入推进医疗卫生资源整合。落实公共卫生服务均等化项目。完成一批医疗卫生投资项目。落实承办第十二届全运会赛事工作，启动建设“十二运”体育场馆。完善政策体系，促进人口长期均衡发展。加强统计信息化建设，完成人口普查任务。加强残疾人事业服务体系建设。认真做好人民防空、民族宗教、外事侨务、国家安全、对台、质量、文史、保密、扶贫、地方志、双拥、优抚、老龄、地震、气象等各项工作。

　　 全力以赴办好以下十件实事：一是实现4.9万人稳定就业。返溪高校应届毕业生就业率力争达到95%以上。二是统筹推进新型农村和被征地农民的养老保险工作。在沈溪新城、太子河新城和桥北新城启动城乡统筹、农民市民化试点。三是启动实施居民医疗保险门诊统筹。全力推进困难企业在职职工参加基本医保。四是调整失业保险、工伤、工亡待遇标准和提高企业退休人员养老金。五是对12万弱势群体开展好法律援助年活动。六是启动建设一批幼儿园、养老公寓。七是完成主城区110条“摸黑路”整治。八是新增廉租房实物配租4300户。九是建成3万个社会治安动态视频监控点。十是完成30处农村饮水安全工程。

　　 (九)切实抓好民主法制和社会稳定等工作。坚持依法治市，启动“六五”普法工作。认真落实市人民代表大会及其常委会的决议、决定，坚持向市人大常委会报告工作制度，自觉接受人大法律监督和工作监督。积极支持市政协履行政治协商、民主监督和参政议政职能，主动接受市政协的民主监督。坚持向各民主党派、工商联和无党派人士通报工作制度，坚持政府部门与各民主党派、工商联对口联系制度。认真负责地办好人大建议和政协提案。积极支持国防和部队建设，深入开展双拥共建活动。切实加强基层政权建设，做好第八届社区居委会换届选举。全面推进精神文明建设。强化社会治安综合治理。加大治安巡逻防控力度，严厉打击各种违法犯罪活动。深入开展安全生产大检查和重点行业专项整治，加强消防、交通以及易燃易爆物品管理，推动安全生产各项工作落实。创新信访工作体制机制，拓展信访工作职能，推进信访问题解决和矛盾隐患化解。

　　 （十）进一步加强政府自身建设。历史的重任，时代的挑战，人民的期待，对政府工作提出了更高要求。我们要全面理解和落实坚持科学发展很重要，坚持抢抓机遇很重要，坚持改革开放很重要，坚持脚踏实地很重要，坚持政通人和很重要。要以创新、规范、高效、廉洁为着力点，切实加强政府自身建设。要与时俱进，提高开拓创新能力。主动顺应时代发展潮流，坚持自觉学习，提高政治素质、理论水平和行政能力。善于运用创新思维、改革思路、市场经济办法，解决好改革、发展、稳定中的难点问题和人民群众关注的热点问题。要规范行为，提高依法行政能力。认真贯彻落实《国务院关于加强法治政府建设的意见》，按照法定权限和程序行使职权、履行职责，提高政府规章质量，强化行政执法监督。健全政府依法决策机制，完善社会公示、重大决策听证和专家咨询制度。要真抓实干，提高为民办事能力。各级政府、各个部门的领导干部要大力弘扬坚定信心、坚韧实干、坚持创新、坚决一流的“药都精神”，率先垂范，靠前指挥，事必躬亲，亲历亲为，百折不挠，一抓到底。要勇于负责，雷厉风行，动真碰硬，敢抓敢管，务求实效。要抓紧每一天，抓好每件事，抓实每个项目。多做群众急需的事，多做群众受益的事，多做打基础利长远的事。做好全市绩效考评工作，推进机关效能建设。要从严治政，提高廉洁自律能力。从市政府领导班子带头做起，严格执行廉洁自律各项规定。强化监察、审计对重大工程、产权交易、土地出让、政府采购等重点领域的管理和监督，严肃查处各类违法违纪案件，坚决纠正部门和行业不正之风。要奋发有为，始终保持昂扬向上的精神状态、拼搏进取的工作激情，组织和带领全市人民抓住前所未有的机遇，开创前所未有的事业。

　　 各位代表、同志们，昂首“十二五”、建设新本溪是历史和人民赋予的重任。让我们在省委、省政府和中共本溪市委的正确领导下，坚定信心，满怀激情，团结一心，创新实干，为全面完成各项工作任务而努力奋斗！
